# Effect of Wall Texture on Perceptual Spaciousness of Indoor Space

**DOI:** 10.3390/ijerph17114177

**Published:** 2020-06-11

**Authors:** Chong Wang, Wei Lu, Ryuzo Ohno, Zongchao Gu

**Affiliations:** 1 School of Architecture and Art, Dalian University of Technology, Dalian City 116024, China; wchonel@mail.dlut.edu.cn (C.W.); gzc1001@dlut.edu.cn (Z.G.); 2 Tokyo Institute of Technology, Tokyo 152-8550, Japan; rohno@ohno-lab.jp

**Keywords:** wall texture, perceptual spaciousness, indoor space, VR technology, ME (magnitude estimation) method

## Abstract

As the main place of people’s daily activities, indoor space (its size, shape, colors, material and textures, and so on) has important physical, emotional and health-based implications on people’s behavior and quality of life. Material texture is an integral part of architectural environment perception and quality evaluation, but the effect of material texture on perceptual spaciousness lacks the support of experimental data. This research examined the effects between different wall textures on the observer’s perception of spaciousness in indoor space, the influence of wall texture changes in different room sizes, and how the associational meaning of texture affects the degree of influence of wall texture on the spaciousness of indoor space. By using VR technology and the magnitude estimation (ME) analysis method, the authors found that the effect of wall texture on perceptual spaciousness varies depending on the wall material, and the textural effect is affected by room size. The perception of spaciousness is influenced by the observer’s associational meaning of material texture, and the influence of associational meaning of material texture varies contingent on the room size. In relatively small rooms, the objective aspect (such as hardness, surface reflectivity, texture direction and texture depth) of the wall texture has a significant impact on perceived space. In contrast, the effects of subjective aspects (such as affinity and ecology) become more pronounced in relatively larger rooms. This research makes up for the lack of material texture research in perceptual spaciousness, and provides a new way for the designer to choose materials for the design of a spatial scale.

## 1. Introduction

With the improvement of people’s quality of life, indoor environmental quality (IEQ) is concerned not only with the traditional physical parameters (e.g., noise, light and temperature) but also the psychological impact of multisensory elements (e.g., visual settings). Given that the interface of interior space is composed of building materials, the texture of the material is an essential aspect of the building environment perception and quality evaluation. Particularly due to the impact of Covid-19, more people are staying in closed indoor environments much longer, which makes the quality of the indoor environment more potent in affecting people’s mental health and wellbeing. Improving the perception of space scale through the design of material texture would thus yield more comfort and satisfaction.

### 1.1. Relevance of Architecture

In the field of architectural design, the vital role of material texture has been recognized for decades. In the Bauhaus, Moholy-Nagy [1] emphasized the importance of material experience in his design teaching (including basic knowledge of material characteristics, processing technology and tools), and introduced “sensory training”, in which students were trained in experiments with systematically arranged textures. Rasmussen [2] proposed the important role of material texture in the design of a series of concrete design cases for Le Corbusier. Norberg - Schulz [3] considered that “the boundary defines a domain in relation to its surroundings,” while texture offers “knowledge of the general character of the district.” Ashihara [4] and Hesselgren [5] emphasized the role of material texture in architectural design, but failed to mention how to perceive or evaluate the differences in texture. Up to now, theoretical research on the texture of building materials still focuses on the fundamental properties of the texture of materials [6], their composition technology [7] and the application of visual expression [8]. Huang [9] argues that there is still no scientific theory to explain the essential relationship between material texture and space, particularly the relationship between material and scale perception.

In terms of the application of material texture in architectural design, existing theories have done much work in the subjective description of space emotion and atmosphere, such as perceptual performance [10], experience design [11] and application performance [12]. However, these descriptions have not been strongly supported by objective experimental data. As an important part of architectural design, from Ergonomics to Environment–Behavior Studies, the research of space scale perception focuses on the impact of the change of actual space size on people, such as the psychophysical experiment of Komiyama et al. [13] on the sense of a room’s volume and spaciousness. In the research on the influence of building materials on spatial scale perception, designers pay more attention to the visual effect of color [14], but lack the quantitative research on the influence of material texture on spatial scale perception.

### 1.2. Relevance of Psychology

The focus on texture in psychology can be attributed to J.J. Gibson’s emphasis on the importance of texture in the perception of the visual world [15]. While Gibson discussed the visual perception of three-dimensional space based on the layout of textured surfaces, he did not mention the influence of texture on a room’s spaciousness [16]. The development of “haptic” research in psychology explains the internal relationship between “visual world” and texture, which transforms the visible material texture into tactile information in consciousness. Numerous studies have confirmed the influence of haptic sensations on the perception of scale, shape, location and distance in space [17,18,19,20]. In previous studies, the author has done experiments on the effect of texture on the perception of material size, and found that the roughness and hardness of texture will affect the perception of the size of the touched material [21]. They then speculated that the texture would also have a particular impact on the perception of spatial scale in three-dimensional space, and confirmed the existence of this phenomenon through experiments [22].

### 1.3. The Goal of This Paper

In order to make up for the lack of material texture research in spatial scale perception, this paper aims to indicate how the room’s wall texture influences human spatial scale perception by finding out the elements of texture causing the influences, as well as the principle revealing how it works. The following hypotheses will be verified in this article:

(1) Wall texture affects the observer’s perception of spaciousness in indoor space.
(2) The influence of wall texture changes according to the room size (observation distance).
(3) The degree of influence of wall textures on the spaciousness of indoor space is dependent on the associated meaning of texture (material).

Based on the above hypotheses, two related articles were retrieved on the web of science. Bokharaei et al. [23] assessed the perceived spaciousness and preference for a destination space in relation to six attributes (size, lighting, window size, texture, wall mural and amount of furniture) and the space experienced before it. In the experiment, it has been verified that the texture in different directions (horizontal and vertical) does not affect perceived spaciousness. Simpson et al. [24] examined that the dominant scale of a wallpaper pattern impacts subjective spaciousness judgments, and alters action-based measures of a room’s size. Unlike the previous research object’s one-sided understanding of texture, or being limited to interior decoration materials, the texture of commonly used building materials is taken as the research object in this paper, and comprehensively analyses all elements of texture. Although the experimental environment is virtual indoor space, it will be extended to the research of outdoor building volume and street space in the future, which will play a more guiding role in the space scale design of architectural designers.

For this purpose, a virtual experiment space was built with VR technology, and the method of magnitude estimation (ME) was introduced in the analysis. In Section 2, using various wall materials in a fixed spatial scale, different wall textures were compared on how they affected the observer’s perception of spaciousness in indoor space. In Section 3, the scale of space in the experiment was altered to compare the effects of material on spatial perception on different spatial scales. In Section 4, an Architectural Material Texture Description Scale is introduced to find out the elements of texture that influence the spatial scale perception.

## 2. Influence of Wall Texture on Perceptual Spaciousness (Experiment 1)

The first experiment was conducted to examine the influence of wall texture on perceived spaciousness. In this test, participants were asked to rate the size of virtual rooms with nine different wall textures. The respondents were made up of 34 college students (19 male and 15 female) aged 17–33, studying architecture, physics, humanities, economy, chemical engineering and many other disciplines, who volunteered to be part of the experiment.

### 2.1. Method

**Figure 1:**
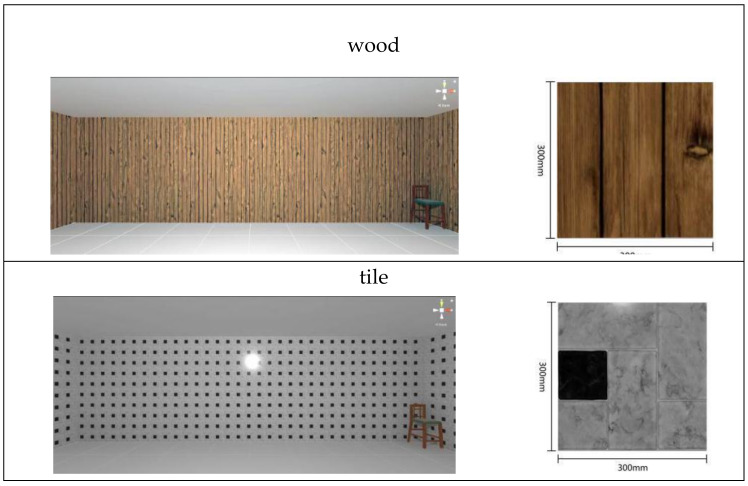

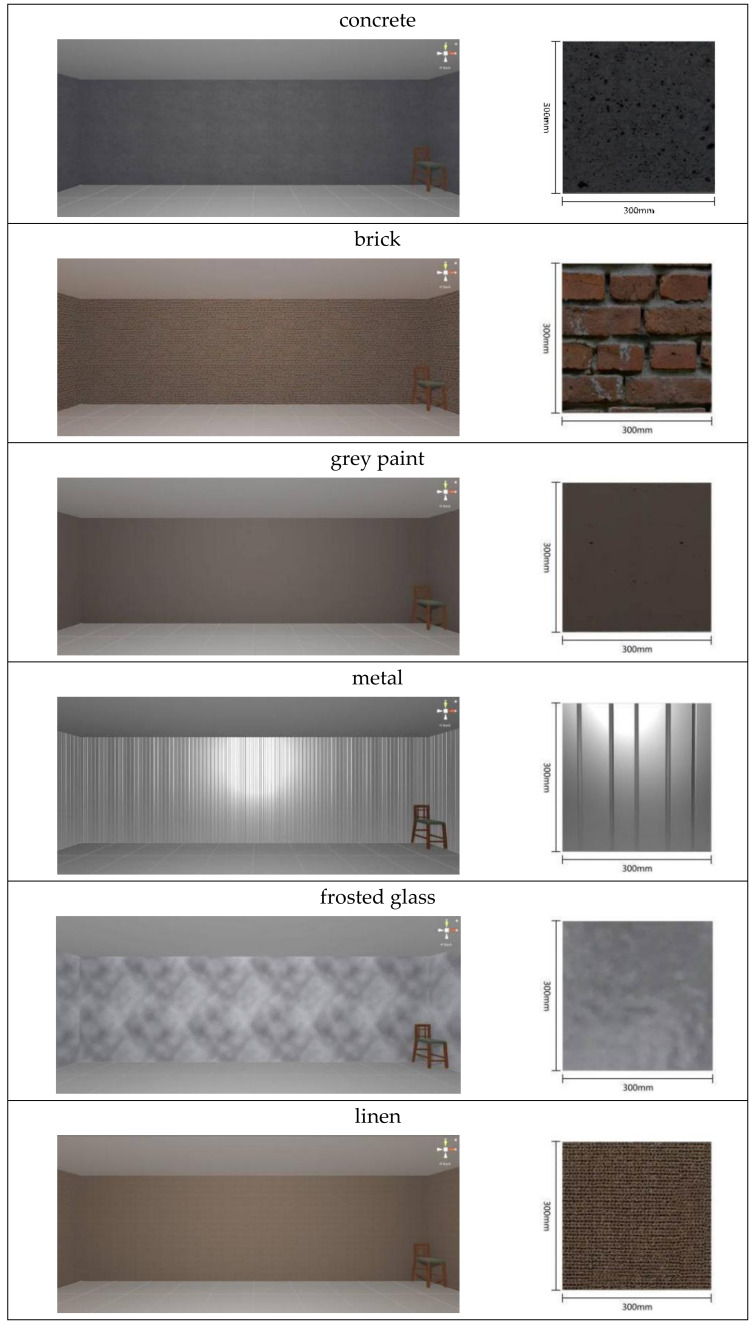
Relative stimuli for Experiment 1.

As the standard stimulus, a room with white walls (no texture) was built in VR space, measuring 10 m in width, 10 m in depth and 3 m in height. The wall finish of the standard stimuli was replaced with eight different textures based on a previous study [25] in creating the relative stimuli, as shown in Figure 1. A chair was installed in the VR room to provide realistic cues as to the absolute size of the space.

**Figure 2:**
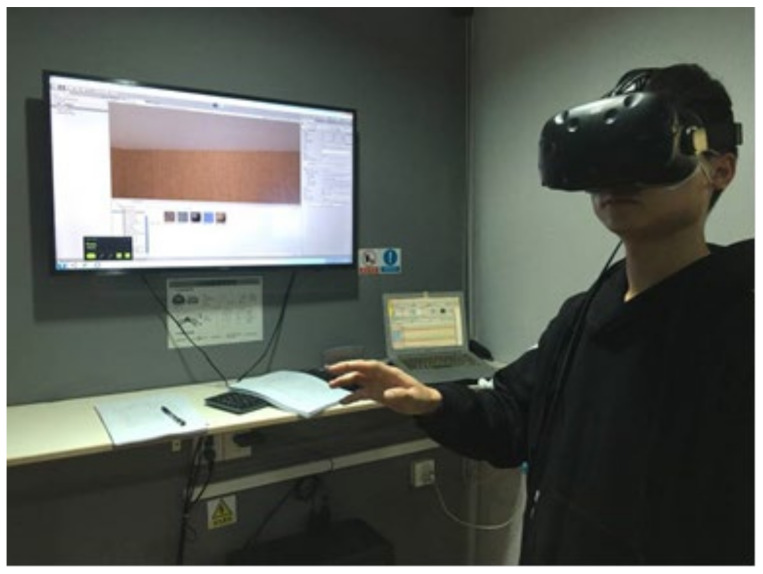
A participant Wearing VR Headset.

The participants were asked to wear a VR headset in order to observe the standard stimulus freely for 15 s. They were then asked to examine the relative stimulus for 15 s (see Figure 2). To minimize disturbances in experiments, the luminance in this VR environment was constant, while other people kept quiet in the lab. Because of the influence of visual persistence, in order to avoid the aftereffect from the previous image, a ten-second interval (blank screen) was inserted between stimuli. Participants were asked to score the spaciousness (room size) of the relative stimulus using the ME method. In this method, the participant scores the relative stimulus in comparison with the standard stimulus, which has a value of 100. The participants were only asked to rate the room size during the experiment, and no other information was provided before the experiment to avoid cognitive tendency.

### 2.2. Calculating the Expansion Ratio

In order to obtain the numerical value gauging the influence of the wall texture, the expansion ratio (ER) is defined by the following equation

ER (r, s) = 1/100 ME(r, s)(1)

where ER (r, s) is the expansion ratio of the relative stimulus (r) against the standard stimulus (s), and ME (r, s) is the number assigned to the relative stimulus (r).

### 2.3. Results 

Since the results show a normal distribution for all relative stimuli, the average value of ER (r, s) can be used to characterize the relative influence of wall textures on perceptual spaciousness. Table 1 shows the average value of ER (r, s) for the eight tested wall textures. Results from the Mann–Whitney U test indicate that the difference between the standard stimulus and all the relative stimuli are statistically significant (*p* < 0.01). Each room finished with a texture was perceived to be significantly smaller than the standard stimulus (no texture). Comparative analysis of the differences in the ER value among the relative stimuli was conducted using the Kruskal–Wallis ANOVA test. The results show the room with the wood wall was perceived to be significantly smaller than the rooms with a metal wall (*p* < 0.05), frosted glass wall (*p* < 0.05) and linen wall (*p* < 0.01), but there was no significant difference in the paired comparison of other materials. This suggests that an individual’s perceptual spaciousness of a room can be influenced by its wall texture.

### 2.4. Discussion

The average ER value of the eight materials are all less than 1, which illustrates that perceptual spaciousness with white walls always seems larger than that with eight other types of material texture for the equal scale, among which the perceptual spaciousness of wood wall is the most narrow, and that of the linen wall is the most spacious.

The results from Experiment 1 suggest that for similarly sized rooms, a textured walled room is perceived as less spacious compared to a room without a wall texture. Furthermore, the impact of wall texture on perceptual spaciousness differs for varying wall materials.

## 3. Influence of Wall Texture on Perceptual Spaciousness in Different Room Size (Experiment 2)

Since wall texture can appear differently for varying observation distances, its effect on the perception of spaciousness could also vary for different room sizes. To examine the effect of room size on the textural effect, the authors conducted an experiment using virtual rooms of six different sizes. Thirty-two (18 male and 14 female) college students aged 17–33 voluntarily participated in this experiment.

### 3.1. Method

In addition to the virtual room used in Experiment 1, five other rooms of varying sizes were created in VR space. The ceiling height for each room was kept at 3 m. The room sizes were determined based on the dimensions of standard function rooms in architecture (see Table 2). Similar to Experiment 1, a white-walled room (no texture) was used as the standard stimulus for each room size. Three textures (materials) were selected as relative stimuli: wood, ceramic tiles and linen (Table 3). The general procedure for Experiment 2 is similar to the methodology in Experiment 1. If the participant participated in both experiments, there should be at least half an hour’s rest to avoid misjudgment due to fatigue.

### 3.2. Results 

Table 4 provides a summary of the average value of the ER for the three wall textures. Since the results show normal distribution for all relative stimuli, the average value of ER (r, s) can be used to characterize the relative influence of the wall textures. All of the textures analyzed show statistically significant differences (*p* < 0.01) from the standard stimulus (no texture), except for the linen wall in the 30 m × 30 m room.

**Figure 3:**
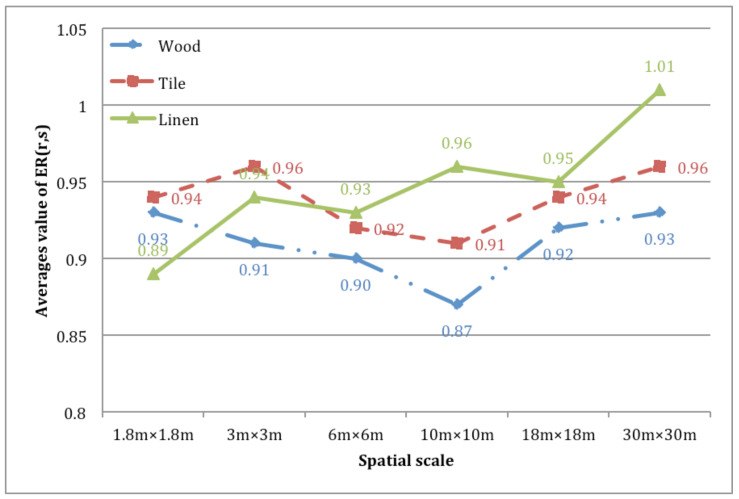
Average values of ER.

Figure 3 shows the change in average value of ER for the wall textures with increasing room size. For each wall texture, the Kruskal–Wallis ANOVA test was applied to examine the differences between rooms at varying dimensions. Statistically significant differences were found in the rooms with wood walls (one case) and with linen walls (three cases), as shown in Table 5.

### 3.3. Discussion

In Experiment 2, the results indicate that the effect of wall texture on perceived spaces varies according to the room size. 

As shown in Figure 3, the ER value of the room with linen walls linearly increases as the room dimensions increase, and it reaches the value of 1 in the largest room (30 m × 30 m). This means that the effect of wall texture on the observer’s subjective judgment of space diminishes as the room becomes bigger. At a particular room size, the perceptual spaciousness of the textured room would be similar to the room with white walls (no texture). This result can be explained by the relationship between the observation distance and the perceived surface roughness (size of texture elements). For smaller rooms, participants can perceive wall texture clear enough to have textural effect, but for larger rooms, texture tends to dissipate, particularly for linen walls.

For wood walls, the ER value for the mid-range-sized room (10 m × 10 m) is lower compared to the values of smaller and larger rooms. This can be as a result of the relationship between the observation distance and perceived surface roughness (size of texture elements). Ohno et al. [26] suggest that there is an optimal scale range of visual aggregated elements that can be perceived as “texture” in a psychophysical experiment. This means a sense of visual texture pattern becomes more evident in a particular range of observation distance. For wood wall rooms, the textural impression was strongest in the medium-sized room (10 m × 10 m), resulting in the textural effect on spaciousness being most evident.

## 4. Influence of Associational Meaning of Material Texture on Perceptual Spaciousness (Experiment 3)

The results from Experiments 1 and 2 reveal that the textural effect on perceptual spaciousness varies depending on the type of material texture and room size. These variations are mainly caused by the visibility of texture elements and the effectiveness of texture patterns. However, texture perception not only handles a surface’s visual pattern, but also evokes the associational meaning of building materials. Therefore, how the building materials’ associational meaning influences an individual’s perceptual spaciousness should be examined. For Experiment 3, the authors hypothesize that perceptual spaciousness is affected by the observer’s associational meaning for a given material texture. The participants’ ratings for each material texture were used in the Pearson correlation analysis, together with the ER values obtained from Experiments 1 and 2 in verifying the hypothesis.

### 4.1. Method

In order to find the proper terms to describe the associational meaning of material texture, the authors reviewed 80 references describing various properties of material texture [12,18,27,28,29,30,31,32,33,34,35,36,37,38,39,40]. 32 relevant semantic scales (bipolar adjective pairs) in reference to literature [41] were selected. The authors then classified the references into two categories: the objective aspect, which can be further subdivided into the physical property and surface property; and the subjective aspect, which comprises utility evaluation and aesthetic evaluation. 

All the participants from Experiments 1 and 2 were shown each of the virtual rooms used in the previous experiments. They were then asked to rate the scene using 32 semantic scales (bipolar adjective pairs), as summarized in Table 6. In order to quantify the participants’ feelings on a given material texture, a five-level scoring system was used: 1 for extremely left-end adjective, to 5 for extremely right-end adjective. 

### 4.2. Results 

The Pearson correlation analysis was used to calculate the correlation coefficient between the ER values of wall textures and the scores from the 32 semantic scales. When the cut-off value of the correlation coefficient was set to 0.4 (weak correlation), nine dependent relations were found, and are summarized in Table 7. The results suggest that associational meaning on some material textures can have a significant effect on people’s perception of spaciousness. 

In a small-sized room (1.8 m × 1.8 m), such as a small kitchen or bathroom, people who identify wood walls as hard or reflective tend to perceive less space, while those who consider linen walls as non-directional tend to perceive more space. In a large living room or small office (6 m × 6 m), people who perceive ceramic tile walls’ texture depth as flat tend to perceive less space. In a classroom or medium-sized meeting room (10 m × 10 m), people who consider wood walls as complex, those who think frosted glass as elastic, those who attribute metal walls as inelastic or crude and those who identify concrete walls as being natural are more inclined to perceive less space. 

### 4.3. Discussion

The results from Experiment 3 indicate that for specific cases, the observer’s view on a given material affects perceptual spaciousness. As discussed in the previous section, the perceptual impact of wall texture varies depending on the room size (observation distance). Similarly, the influence of the associational meaning of material texture is affected by the room size. In larger rooms (18 m × 18 m and 30 m × 30 m), no significant correlation (r < 0.4) between the participants’ semantic scores and wall textures was found. For medium (10 m × 10 m) and small-sized rooms, the authors found several semantic scales to have high correlation with some material textures. Moreover, the objective aspects were found to have substantial influence over relatively smaller rooms, while the subjective aspects (aesthetic evaluation) are influential in relatively larger rooms. 

One possible explanation for the results is that the observation distance can have a considerable effect on the sharpness of the observer’s vision. In small rooms, observers acquire information mainly from physical attributes (hardness) and surface properties (reflectivity, texture direction). In mid-sized rooms, longer observation distances can reduce textural details of materials, and therefore the impact of the subjective aspects (naturality, delicacy) responsible for the room’s global impression becomes more substantial. In large rooms, as the wall is too far away, the observer cannot accurately evaluate the texture elements, and the description of texture depends more on the understanding of the wall material in memory, so what has a greater impact on perceptual spaciousness is the inherent impression of the wall material.

## 5. Conclusions

In this paper, a virtual space was built by VR technology to test the effect of wall texture on perceptual spaciousness and determine the effect differences between nine wall textures on perceptual spaciousness and its trend under different spatial scales. The internal elements of these phenomena are analyzed using the Architectural Material Textural Semantic Descriptive Scale, which is used to explain the mechanism of the effect of wall texture on perceptual spaciousness. This study provides a new approach for designers to choose materials for their design of spatial scale.

The main highlights of the study are as follows: (1) A room with textured walls is perceived as less spacious than an untextured room of similar size. (2) The effect of wall texture on perceptual spaciousness varies depending on the wall material. (3) Textural effect is affected by room size (observation distance). A fine texture, such as linen walls, reduces textural effect when the observation distance is increased. For rough or clear-pattern textures, such as wood walls, a particular range of observation distance results in distinct textural effects. (4) The perception of spaciousness is influenced by the observer’s associational meaning of material texture. (5) Moreover, the influence of the associational meaning of material texture varies contingent on the room size. In relatively small rooms, the objective aspects of the associational meaning have a significant impact on perceived space, while the effects of subjective aspects (aesthetic evaluation) become more pronounced in relatively large rooms. 

Note that the above results have been extracted under VR laboratory situations, and the stimuli used in the experiment had been limited. Nevertheless, as an initial attempt to scientifically understand the textural experience, the present study provides a preliminary reference for various applications of building materials in space design. In our follow-up study, experimental data would be supplemented, and other determinants (such as the color, pattern and cleaning properties of materials, the age and regional cultural difference of participants) would be further analyzed.

The findings of this study can be used as reference for designers when selecting the type of interior materials to use in order to provide a more comfortable and healthier living environment for users. This study would be particularly useful in instances where perceived spaciousness of indoor space is of primary concern. As to whether the effect of material texture on perceptual spaciousness is equally effective outdoors, the researchers will take building monomer and street interface as an example for further research in the future.

## Figures and Tables

**Table 1:** Result of Experiment 1 using the Mann–Whitney U test.

Wall Textures	Average Value of ER
Wood	0.87 **
Ceramic tile	0.91 **
Concrete	0.92 **
Brick	0.92 **
Grey paint	0.92 **
Metal	0.94 **
Frosted glass	0.94 **
Linen	0.96 **

** significant at the 0.01 level.

**Table 2:** Tested virtual rooms: dimensions and common function.

**Room Dimensions**	1.8 m × 1.8 m	3 m × 3 m	6 m × 6 m	10 m × 10 m (Experiment 1)	18 m × 18 m	30 m × 30 m
**Common Function**	Bathroom, Closed kitchen	Bedroom	Living room, Office	Classroom, Meeting room	Lobby, Lecture hall	Theatre, Auditorium

**Table 3:** Relative stimuli for Experiment 2.

Scale	Wood	Ceramic Tile	Linen
1.8 × 1.8 m^2^	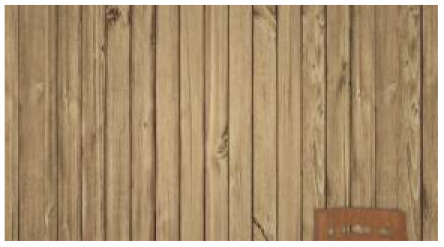	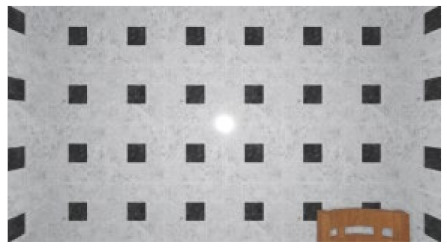	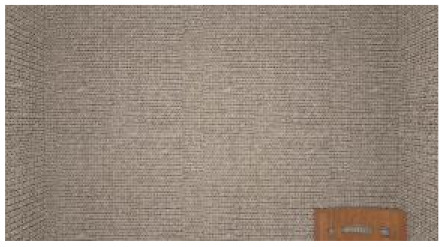
3 × 3 m^2^	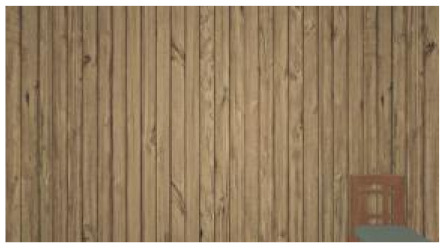	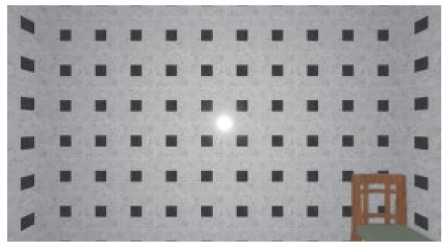	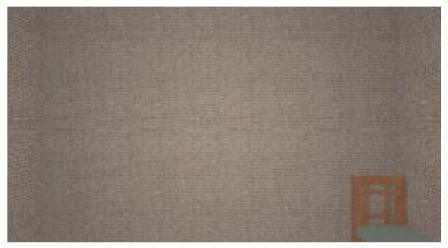
6 × 6 m^2^	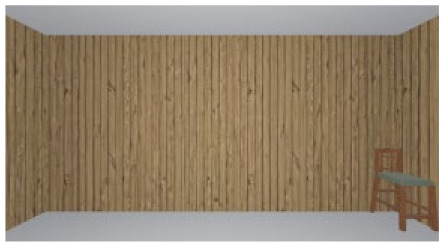	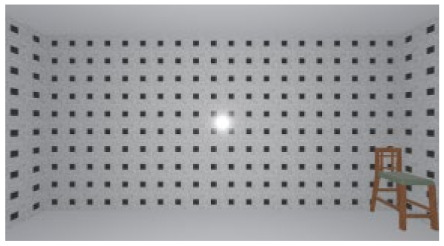	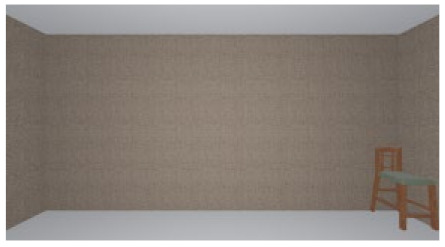
10 × 10 m^2^	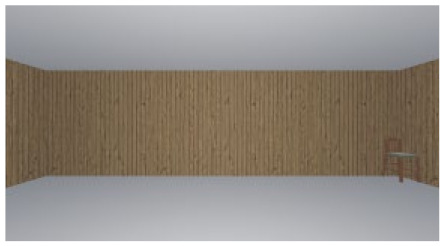	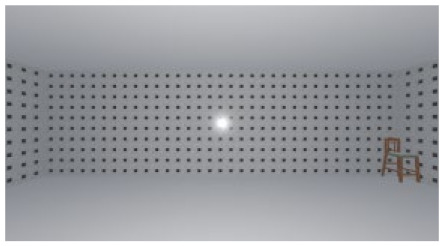	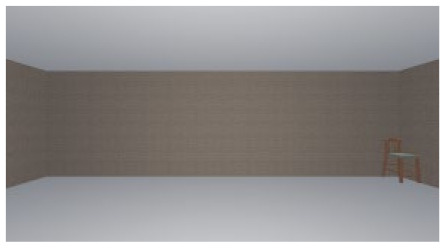
18 × 18 m^2^	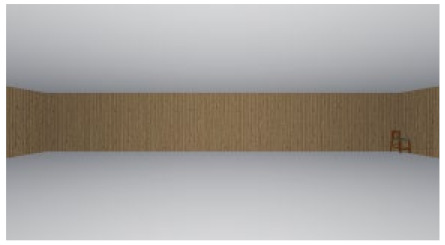	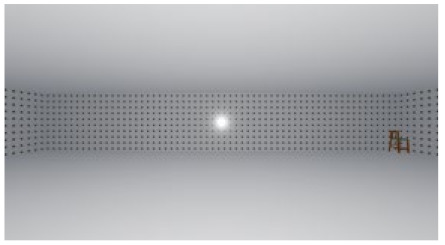	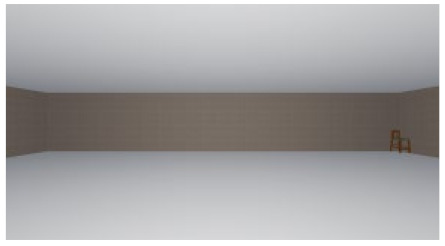
30 × 30 m^2^	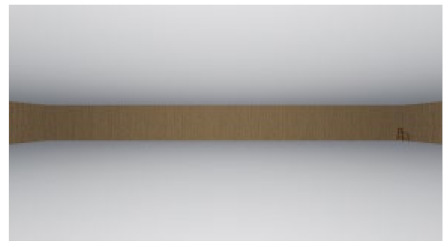	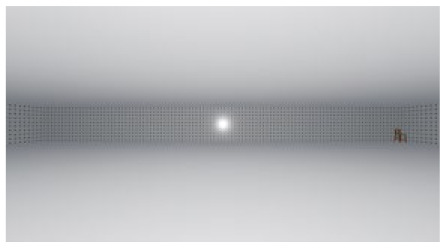	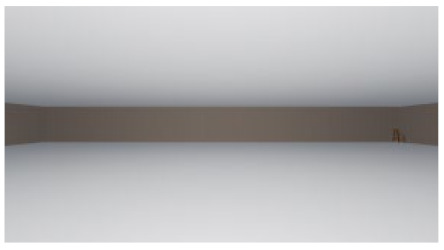

**Table 4:** Mann–Whitney U test Results for Experiment 2.

Room Dimensions	Wall Textures		
	Wood	Ceramic Tile	Linen
1.8 m × 1.8 m	0.93**	0.94**	0.89**
3 m × 3 m	0.91**	0.96**	0.94**
6 m × 6 m	0.90**	0.92**	0.93**
10 m × 10 m	0.87**	0.91**	0.96**
18 m × 18 m	0.92**	0.94**	0.95**
30 m × 30 m	0.93**	0.96**	1.01

** significant at the 0.01 level.

**Table 5:** Kruskal–Wallis ANOVA test of ER values between same wall textures at varying room sizes.

Wall Textures	Room Dimensions	ER Values	Z	*p*
Wood	10 m × 10 m	0.87 ± 0.11	−2.323	0.020
	30 m × 30 m	0.93 ± 0.10		
Linen	1.8 m × 1.8 m	0.89 ± 0.16	−2.338	0.019
	10 m × 10 m	0.96 ± 0.12		
	1.8 m × 1.8 m	0.89 ± 0.16	−3.461	0.001
	30 m × 30 m	1.01 ± 0.09		
	6 m × 6 m	0.93 ± 0.09	−3.126	0.002
	30 m × 30 m	1.01 ± 0.09		

**Table 6:** Semantic Descriptive Scales for Material Texture in Architecture.

**Objective Aspect**			
Physical Property		Surface Property	
Hardness	Soft - Hard	Surface Roughness	Smooth - Rough
Strength	Fragile - Sturdy	Morphological Integrity	Complete - Defective
Elasticity	Elastic - Inelastic	Surface Reflectivity	Reflective - Non-Reflective
Viscosity	Viscous - Not Viscous	Texture Form	Clear Texture - Blurred Texture (No Texture)
Density	Porous - Dense	Texture Direction	Non-directional - Directional(Transverse/Vertical/Tilt/Cross)
Temperature	Warm - Cold	Texture Density	Intensive - Scattered
Humidity	Dry - Wet	Texture Depth	Bumpy - Flat
Weight	Heavy - Light	Symmetry	Regular - Irregular
Transparency	Transparent - Opaque	Complexity	Simple - Complex
**Subjective Aspect**			
Utility Evaluation		Aesthetic Evaluation	
Comfort	Comfortable - Uncomfortable	Time Sense	Traditional - Modern
Value	Plain - Gorgeous	Ecology	Natural - Artificial
Function	Practical - Decorative	Order	Neat - Dirty
Feasibility	Durable - Temporary	Affinity	Elegant - Crude
Safety	Safe - Dangerous	Vitality	Brand New - Stale
Scope of Application	Indoor - Outdoor	Stability	Stable - Unstable
		Charm	Exquisite - Flawed
		Favorite	Annoying - Favorite

**Table 7:** Correlation between ER Value of Wall Textures and Score on Semantic Scales.

Visual Stimuli		Semantic Scales		Correlation Coefficient
Room Dimensions	Wall Textures	Category	Bipolar Adjective Pairs	
1.8 m × 1.8 m	Wood	Objective _ Physical	Soft - Hard	−0.48
		Objective _ Surface	Reflective - Non-Reflective	0.40
	Linen	Objective _ Surface	Non-Directional - Directional	0.41
6 m × 6 m	Ceramic tile	Objective _ Surface	Bumpy - Flat	−0.40
10 m × 10 m	Wood	Objective _ Surface	Simple - Complex	−0.42
	Frosted glass	Objective _ Physical	Elastic - Inelastic	0.42
	Metal	Objective _ Physical	Elastic - Inelastic	−0.40
		Subjective_ Aesthetic	Elegant - Crude	−0.42
	Concrete	Subjective_ Aesthetic	Natural - Artificial	0.58
